# Supplementary material for: Ongoing Efforts to Improve Antimicrobial Utilization in Hospitals among African Countries and Implications for the Future
Source: Antibiotics (Basel). 2022 Dec 15;11(12):1824. doi: 10.3390/antibiotics11121824 (PMC9774141; doi:10.3390/antibiotics11121824)
Supplement: Supplementary file 1 [file antibiotics-11-01824-s001.zip › antibiotics-2071635-supplementary.pdf]

Supplementary Material

Ongoing Efforts to Improve Antimicrobial Utilization in Hospitals among African Countries and Implications for the Future

Zikria Saleem [1], Brian Godman [2,3,4,*], Aislinn Cook [5,6], Muhammad Arslan Khan [7], Stephen M. Campbell [4,8,9], Ronald Andrew Seaton [10,11], Linda Siachalinga [12], Abdul Haseeb [13], Afreenish Amir [14], Amanj Kurdi [2,4,15,16], Julius C. Mwita [17], Israel Abebrese Sefah [18], Sylvia A. Opanga [19], Joseph O. Fadare [20,21], Olayinka O. Ogunleye [22,23], Johanna C. Meyer [4,24], Amos Massele [25], Dan Kibuule [26], Aubrey C. Kalungia [27], Moyad Shahwan [3,28], Hellen Nabayiga [29], Giuseppe Pichierri [30] and Catrin E. Moore [5]

1 Department of Pharmacy Practice, Faculty of Pharmacy, Bahauddin Zakariya University, Multan 60000, Pakistan
2 Department of Pharmacoepidemiology, Strathclyde Institute of Pharmacy and Biomedical Sciences,
University of Strathclyde, Glasgow G4 0RE, UK; amanj.baker@strath.ac.uk
3 Centre of Medical and Bio-Allied Health Sciences Research, Ajman University, Ajman 346, United Arab Emirates
4 Department of Public Health Pharmacy and Management, School of Pharmacy, Sefako Makgatho Health Sciences University, Molotlegi Street, Garankuwa, Pretoria 0208, South Africa
5 Centre for Neonatal and Paediatric Infection, St. George’s University of London, London SW17 0RE, UK
6 Health Economics Research Centre, Nuffield Department of Population Health, University of Oxford,
Oxford OX1 2JD, UK
7 The Indus Hospital, Bedian Road, Lahore Plot no. 779-802, Pakistan
8 Centre for Epidemiology and Public Health, School of Health Sciences, University of Manchester,
Manchester M13 9PL, UK
9 NIHR Greater Manchester Patient Safety Translational Research Centre, School of Health Sciences,
University of Manchester, Manchester M13 9PL, UK
10 Queen Elizabeth University Hospital, Govan Road, Glasgow G51 4TF, UK
11 Scottish Antimicrobial Prescribing Group, Healthcare Improvement Scotland,
Delta House, 50 West Nile Street, Glasgow G1 2NP, UK
12 College of Pharmacy, Yeungnam University, Daehak-Ro, Gyeongsan, Gyeongbuk 38541, Republic of Korea
13 Department of Clinical Pharmacy, College of Pharmacy, Umm Al-Qura University, Makkah 24382, Saudi Arabia
14 Department of Microbiology, Armed Forces Institute of Pathology National University of Medical Sciences, Rawalpindi 46000, Pakistan
15 Department of Pharmacology, College of Pharmacy, Hawler Medical University, Erbil 44001, Iraq
16 Center of Research and Strategic Studies, Lebanese French University, Erbil 44001, Iraq
17 Department of Internal Medicine, Faculty of Medicine, University of Botswana, Private Bag 0713 UB, Gaborone , Botswana
18 Pharmacy Practice Department, School of Pharmacy, University of Health and Allied Sciences,
Volta Region, Ho PMB 31, Ghana
19 Department of Pharmaceutics and Pharmacy Practice, School of Pharmacy, University of Nairobi,
Nairobi P.O. Box 19676-00202, Kenya
20 Department of Pharmacology and Therapeutics, Ekiti State University, Ado Ekiti 362103, Nigeria

[21] Department of Medicine, Ekiti State University Teaching Hospital, Ado Ekiti 360211, Nigeria
[22] Department of Pharmacology, Therapeutics and Toxicology, Lagos State University College of Medicine, Ikeja, Lagos 100271, Nigeria
[23] Department of Medicine, Lagos State University Teaching Hospital, Ikeja 100271, Nigeria
[24] South African Vaccination and Immunisation Centre, Sefako Makgatho Health Sciences University, Molotlegi Street, Garankuwa, Pretoria 0208, South Africa
[25] Department of Clinical Pharmacology and Therapeutics, Hurbert Kairuki Memorial University,
70 Chwaku Road Mikocheni, Dar Es Salaam P.O. Box 65300, Tanzania
[26] Department of Pharmacology & Therapeutics, Busitema University, Mbale P.O. Box 236, Uganda
[27] Department of Pharmacy, School of Health Sciences, University of Zambia, Lusaka P.O. Box 50110, Zambia
[28] Department of Clinical Sciences, College of Pharmacy and Health Sciences, Ajman University,
Ajman 346, United Arab Emirates
[29] Management Science Department, Strathclyde Business School, University of Strathclyde,
199 Cathedral Street, Glasgow G4 0QU, UK
[30] Microbiology Department, Torbay and South Devon Foundation Trust, Lowes Bridge Torbay Hospital, Torquay TQ2 7AA, UK
* Correspondence: brian.godman@strath.ac.uk

1. Prescribing/ Quality Indicators including those for hospitals

Any prescribing/ quality indicator that is instigated within healthcare systems must be within the context of strategies to develop and sustain high-quality, patient safety and patient centred focused health care. This involves “the right care, at the right time, responding to the service users’ needs and preferences, while minimizing harm and resource waste” [124]. Such a focus requires parallel systemic strategies and interventions at the macro-meso-micro level for embedding and strengthening the building blocks to optimise the quality of care [124,255].

Going forward for countries, including Africa, it is necessary to distinguish between physical and workforce structures of health care, actual care given (process) and the consequences of the interaction between individuals and a health care system [outcome] [256,257].

While many different indicators have been used to enhance the quality of antimicrobial prescribing in hospitals across countries [9,19,58,112,185,238,258-260], any indicator proposed must adhere to the attributes of good indicators. Key attributes include having clarity, being feasible, and having easy-to-use reliable and consistent (preferably computerized) tools for valid data collection and management [125-127]. This requires data that are consistently and easily available, which can be a challenge for a number of low- and middle-income with countries. In the case of Africa, countries are at different stages of data collection tools, with current patient documentation still principally paper based. This is likely to change though principally through the NAP imperative to drive down AMR across Africa through improved antibiotic use [70].

Box S1. Potential barriers to instigating ASPs in hospitals and improving future antimicrobial prescribing (adapted from [42,76,128,130,176,185,198,199,227]).

- Necessary infrastructure and available HCPs within hospitals to undertake ASPs

• Adequate knowledge among HCPs in hospitals regarding antibiotics and ASPs as well as the role and value of IPC committees<br>• Lack of manpower and resources within hospitals to provide dedicated education for HCPs and monitor subsequent prescribing following their introduction as well as re-education if needed<br>• Currently poor attitudes among HCPs towards undertaking ASPs in hospitals<br>• Lack of critical leadership currently in hospitals to drive ASPs forward - including the development/ introduction/ monitoring of any guidelines and associated quality indicators<br>• Lack of ASP champions currently in the hospital, including physicians and pharmacists, to drive ASPs forward<br>• Current misuse of antibiotics within the hospital is not well documented providing the necessary stimulus to undertake ASPs<br>• No appropriate guidelines, whether national or local, readily available, accessible and easy-to-use in the hospital to guide HCP prescribing in key areas that can be a focus for ASPs<br>• Currently no ongoing research to identify the extent of any guideline adherence as well as any problems - including a lack of faith in current guidelines, their ready availability and ease-of-use<br>• Lack of up-to-date information regarding antimicrobial resistance patterns within the hospital from reliable sources to appreciably influence empiric prescribing/ be a subject for future ASPs<br>• Adequate facilities and support to undertake diagnostic tests, including culture and sensitivity testing, and speedy results if sent to other higher hospitals, with no prohibitive costs for patients<br>• No one to design ASPs that can be readily undertaken in hospitals which would give confidence for the future<br>• Lack of monitoring of supplies of antibiotics in hospitals or proactively dealing with shortages to minimise any impact of shortages on current ASPs including agreed therapeutic options<br>• Possible financial incentives that currently exist through privately selling antibiotics in hospitals and/ or income based on the cost of antibiotics

ASPs: Antimicrobial Stewardship Programmes; HCPs: healthcare professionals; IPC: Infection, Prevention and Control.

Table S1. Literature review of point prevalence surveys across Africa.

Country	WB Class	Name, date and reference	No. of hosps	PPS method	PPS protocol	Study duration	AMU, n (%)	Most prescribed antibiotic(s) (ATC code, AWaRe and %)	Second most prescibed antibiotic(s)	Third most prescribed antibiotic(s)	Proph. (%)	Treat. (%)	Antimicrobials (per/patient)
Ethiopia	Low	Fentie et.al, 2022 [157]	10	Period	WHO	January 2021	1162 (63.8%)	Ceftriaxone **(W)** *J01DD04* (30.4%)	Metronidazole **(A)** *J01XD01* (15.4%)	Ampicillin **(A)** *J01CA01* (12.1%)	25.5	74.1	1.77
Tanzania	Low	Horumpende et.al, 2020 [159]	3	Period	ECDC	November and December 2016	176 (44%)	Ceftriaxone **(W)** *J01DD04* (28.5%)	Metronidazole **(A)** *J01XD01* (23.9%)	Penicillin **(A)** *J01C* (26.9%)	30.5	52	1.8
Tanzania	Low	Seni et. al, 2020 [64]	6	Period	WHO	December 2019	591 (62.3%)	Ceftriaxone **(W)** *J01DD04* (30.9%)	Metronidazole**(A)** *J01XD01* (22.9%)	Ampicillin-cloxacillin (17.0%)	54.2	36.7	1.72
Uganda	Low	Kiggundu et. al, 2022 [13]	13	Period	WHO	December 2020 to April 2021	794 (73.7%)	Ceftriaxone **(W)** *J01DD04* (37%)	Metronidazole**(A)** *J01XD01* (27%)	Gentamicin **(A)** *J01GB03* (7%)	52.12	47.87	1.5
Congo	LM	Wambale et. al, 2016 [261]	11	Period	Own	October 2014	476 (68%)	Ampicillin **(A)** *J01CA01* (35%)	Gentamicin **(A)** *J01GB03* (13.6%)	Amoxicillin **(A)** *J01CA04* (13.5%)	-	-	1.4
Eswatini	LM	Gwebu et., 2022 [15]	1	14 days	WHO	25 January - 8 February 2021	60 (82.2%)	Amoxicillin **(A)** *J01CA04* (24.3%)	Ceftriaxone **(W)** *J01DD04* (21.6%)	Cloxacillin **(A)** *J01CF02* (9.8%)	40.8	59.2	1.7
Ghana	LM	Labi et. al, 2018 [178]	1	Period	Modified ECDC	February and March 2016	348 (51.4%)	Metronidazole **(A)** *J01XD01* (17.5%)	Amoxicillin-clavulanic acid **(A)** *J01CR02* (13.4%)	Ceftriaxone **(W)** *J01DD04* (12.1%)	39	61.1	1.75
Ghana	LM	Labi et. al, 2018 [262]	10	Period	ECDC	September 2016 to December 2016	506 (70.6%)	3rd generations Cephalosporins *J01DD* (18.5%)	Aminoglycosides *J01G* (17.9%)	2nd generations cephalosporins *J01DC* (12.4%)	23.7	71.4	1.64
Ghana	LM	Afriyie et.al, 2019 [76]	2	2 days	Global PPS	May 2019	-	Penicillin with Beta lactam inhibitor **(A)** *J01CR*	2nd and 3rd generations cephalosporins *J01DC* and *J01DD*	-	-	-	-

Ghana	LM	Bediako-Bowan et al, 2019 [163]	10	Period	ECDC	October to December 2016	382 (70.7%)	Metronidazole**(A)** *J01XD01* (25.6%)	Cefuroxime**(W)** *J01DC02* and ceftriaxone **(W)** *J01DD04* (20.0%)	Amoxicillin **(A)** *J01CA04* and Clavulanic acid *J01CR* (16.7%)	37.7	58.6	1.66
Ghana	LM	Garcia-Vello et al, 2020 [263]	1	-	BNF and BNF Children	January to June 2015	-	Metronidazole **(A)** *J01XD01* (36.5%)	Ceftriaxone **(W)** *J01DD04* (35.3%)	Ciprofloxacin **(W)** *J01MA02* (24.5%)	-	-	-
Ghana	LM	Dodoo et. al, 2021 [77]	1	One day each	Global PPS	July 2019 and January 2020	98 (66.7%) 84 (54.9%)	Penicillin **(A)** *J01C*	Cephalosporins *J01D*	Metronidazole **(A)** *J01XD01*	-	-	2.2 (July 2019) 1.75 (Jan 2020)
Ghana	LM	Amponsah et.al, 2021 [63]	3	3 days	WHO	26th Nov 27th Nov 10th Dec 2019	115 (60.5%)	Penicillin **(A)** *J01C* (48.7%)	Cephalosporins *J01D* (23.5%)	Fluroquinolones *J01MA* (17.4%)	-	52.2	-
Ghana	LM	Ankrah et.al, 2021 [164]	1	3 days	Global PPS	June 2019	527 (53.3%)	Beta lactam inhibitor *J01CR* (17.5%)	Metronidazole **(A)** *J01XD01* (11.8%)	Ceftriaxone **(W)** *J01DD04* (11.5%)	36	64	1.8
Kenya	LM	Momanyi et.al, 2019 [79]	1	One day	Global PPS	April 2017	174 (54.7%)	Penicillin**(A)** *J01C* (46.9%)	Cephalosporins *J01DD* (44.7%)	Aminoglycosides *J01 GB* (26.3%)	29.1	54.2	-
Kenya	LM	Maina et. al, 2020 [186]	14	Period	Global PPS	February to April 2018	1675 (46.7%)	Cephalosporins *J01D* (26%)	Metronidazole **(A)** *J01XD01* (20%)	-	-	-	2.0
Kenya	LM	Okoth et al, 2018 [78]	1	7 days	Global PPS	5th to 12th June 2017	182 (67.7%)	3rd generation Cephalosporins *J01DD* (55%)	Metronidazole **(A)** *J01XD01* (41.8%)	Penicillin **(A)** *J01C* (41.8%)	51	47	1.8
Kenya	LM	Omulo et.al, 2022 [80]	3	24 days	WHO	September 2017 to April 2018	489 (46%)	Amoxicillin Clavulanate **(A)** *J01CA04* Ceftriaxone **(W)** *J01DD04*	Metronidazole **(A)** J01XD01	-	22	-	1.5
Nigeria	LM	Nsofor et, al 2016 [167]	9	Period	ECDC	-	1585 (55.9%)	Penicillins **(A)** (81.9%)	Chloramphenicol (33.3%)	Tetracyclines (33.2%)	11.3 (Surgical)	88.7 (Others)	-

Nigeria	LM	Oduyebo et. al, 2017 [168]	8	Period	Own	April to June 2015	828 (69.7%)	Ceftriaxone **(W)** *J01DD04*	Metronidazole **(A)** *J01XD01*	Ciprofloxacin **(W)** *J01MA02*	38.8	51.2 (10% unspecified)	-
Nigeria	LM	Umar et. al, 2018 [182]	1	Period	Own	January 1 2013 to December 31st 2014	-	Ampicillin **(A)** *J01CA01*- Cloxacillin **(A)** *J01CF02* (44.6%)	Ampicillin **(A)** *J01CA01*- cloxacillin **(A)** *J01CF02* and gentamicin **(A)** *J01GB03* (14.8%)	Penicillin **(A)** J01C and gentamicin **(A)** *J01GB03* (8.3%)	-	-	-
Nigeria	LM	Fowotade et al., 2020 [169]	1	One	Global PPS	15 December 2015	451 (59.6%)	Third generation cephalosporins (23.9%) - mainly ceftriaxone	Penicillin combinations (17.4% - mainly amoxicillin and clavulanic acid	Fluoroquinolones (16.6%)			
Nigeria	LM	Umeokonkwo et. al, 2019 [264]	1	Period	Global PPS	October to November 2017	220 (78.2%)	Ceftriaxone **(W)** *J01DD04* (25.1%)	Metronidazole **(A)** *J01XD01* (24.6%)	-	48.5	51.5	-
Nigeria	LM	Nnadozie et. al, 2020 [114]	1	One day	Global PPS	May 2019	82 (97.6%)	Metronidazole **(A)** *J01XD01* (29.2%)	Ceftriaxone **(W)** *J01DD04* (19.8%)	Ciprofloxacin **(W)** *J01MA02* (14.6%)	57	43	2.71
Nigeria	LM	Chioma, 2020 [265]	1	One day	Own	25th April 2019	34 (85%)	Ceftriaxone **(W)** *J01DD04*	Gentamycin **(A)** *J01GB03*	Cefuroxime **(W)** *J01DC02*	5.9	94.1	-
Nigeria	LM	Aboderin et. al, 2021 [16]	9	Period	WHO	10-27 June 2019	246 (76.6%)	Metronidazole **(A)** *J01XD01* (25.2 %)	Cefuroxime **(W)** *J01DC02* (18.4%)	Ceftriaxone **(W)** *J01DD04* (13.7%)	48.1	38	2.2
Nigeria	LM	Ogunleye et. al, 2022 [14]	1+1	1+1 days	ECDC And Global PPS	November 2019	494 (80.6%)	Metronidazole **(A)** *J01XD01* (32.4%) Metronidazole (19.1%)	Ceftriaxone **(W)** *J01DD04* (27.5%) Ceftriaxone (25.3%)	Amoxicillin+ Clavulanate **(A)** (8.2%) *J01CR02* Amoxicillin +clavulanate (9.3%)	15.82	84.17	1.94
Zambia	LM	Masich et.al, 2020 [266]	1	7 days	WHO	June 2018	88 (60%)	Cefotaxime **(W)** *J01DD01* (35.7%)	Ceftriaxone**(W)** *J01DD04* (22.2%)	Metronidazole **(A)** *J01XD01* (11.1%)	-	-	1.5
Ghana Uganda Zambia Tanzania	Low/ LM	D'Arcy et.al, 2021 [81]	17	Period	Global PPS	May 2021	2169 (50%)	Metronidazole **(A)** *J01XD01* (22.4%)	Co- amoxiclav *J01CR02* (21.6%)	Cefuroxime **(W)** *J01DC02* (11.8%)	-	-	1.7

Botswana	UM	Anand Paramadhas et.al, 2019 [19]	10	30 days	Global and European PPS	3rd may to 14th June 2017	711 (70.6%)	Cefotaxime **(W)** *J01DD01*	Metronidazole**(A)** *J01XD01*	-	38.3	61.7	1.4
South Africa	UM	Dlamini et al, 2019 [180]	1	2 months	ECDC G-PPS adapted	February to march 2017	193 (37.7%)	Beta Lactamase inhibitor *J01CR*	Anti TB agents	-	-	-	1.5
South Africa	UM	Skosana et al, 2021 [171]	18	5 Months	ECDC G-PPS	April to August 2018	1479 (33.6%)	Penicillin **(A)** *J01C*	Amoxicillin **(A)** + inhibitor *J01CR02* (23.1%)	Ceftriaxone **(W)** *J01DD04* (10.7%)	-	-	1.4
South Africa	UM	Skosana et.al, 2022 [61]	18	Period	ECDC/ Global PPS and adapted	April to August 2018	627 (49.7%)	Ampicillin **(A)** *J01CA01* (16.4%)	Gentamycin **(A)** *J01GB03* (10.0%)	Amoxicillin **(A)** *J01CA04*/ enzyme inhibitor (9.6%)	16.4	83.6	1.61

AM: antimicrobial; ATC: Anatomical, Therapeutic, Chemical classification; AMU: Antimicrobial use; A and W: Access and Watch (AWaRe classification); Period: Period of the study if either over several months or within a month with no further details given; Proph: prophylaxis; Treat: treatment; WB Class: World Bank Classification based on [59]; LM: low-middle country; UM: upper-middle country
